# Linking eruptive style with pore network geometry in tephritic/basanitic tephra from the 2021 Tajogaite eruption (Canary Islands, Spain)

**DOI:** 10.1007/s00445-025-01833-0

**Published:** 2025-05-30

**Authors:** Barbara Bonechi, Emily C. Bamber, Margherita Polacci, Fabio Arzilli, Giuseppe La Spina, Elisa Biagioli, Jorge E. Romero, Jean-Louis Hazemann, Richard Brooker, Robert Atwood, Mike Burton

**Affiliations:** 1 Department of Earth and Environmental Sciences, The University of Manchester, Manchester, United Kingdom of Great Britain and Northern Ireland; 2 Institute of Science, Technology and Sustainability for Ceramics (ISSMC), National Research Council (CNR), Faenza, Italy; 3 School of Science and Technology, Geology Division, Camerino, Italy; 4 Istituto Nazionale Di Geofisica E Vulcanologia, Osservatorio Etneo, Catania, Italy; 5 Instituto de Ciencias de La Ingeniería, Universidad de O’Higgins, Rancagua, Chile; 6 Université Grenoble Alpes, CNRS, Grenoble INP, Institut Néel, Grenoble, France; 7 School of Earth Sciences, University of Bristol, Bristol, United Kingdom of Great Britain and Northern Ireland; 8 Diamond Light Source, Harwell Science and Innovation Campus, Harwell, Oxfordshire United Kingdom of Great Britain and Northern Ireland

**Keywords:** X-ray computed microtomography, Vesicle number density, Throat-pore size ratio, Tortuosity, Outgassing, Eruptive dynamics

## Abstract

Investigating the textural properties and 3D geometry of the connected pore network in volcanic products provides insight into magma ascent processes, due to their influence on magma permeability, outgassing efficiency and explosivity. Here, we used X-ray computed microtomography to investigate vesicle textures in tephra from the 2021 Tajogaite eruption (La Palma, Spain) and the relationship between these pore network parameters and eruptive style. We report a 3D dataset of pore network parameters for lapilli clasts collected throughout the eruption, associated with different eruptive styles (ash-rich jets, lava fountains, Strombolian activity). In clasts from Strombolian activity, the lower vesicle number density (VND) and tortuosity factor (*m*) suggests that there are fewer vesicles and that the channels which connect them are less tortuous than in clasts from fountain and ash-rich jet activity, favouring a lower degree of gas–melt coupling and thus, more efficient outgassing. Instead, for clasts of lava fountain and ash-rich jet activity, the higher VND and *m* suggest a higher number of vesicles connected by more tortuous channels, promoting some degree of gas–melt coupling and thus, less efficient outgassing. However, in clasts from ash-rich jets, the presence of narrower channels, as suggested by the lower throat-pore size ratio, favours a greater degree of gas–melt coupling with respect to fountain activity, leading to magma fragmentation. This work highlights the importance of textural and pore network analyses in understanding eruption dynamics, and provides a case study for investigating the interplay between pore network parameters, magma permeability and ascent dynamics for low-viscosity magmas.

**Supplementary Information:**

The online version contains supplementary material available at 10.1007/s00445-025-01833-0.

## Introduction

Eruptive style is governed by interdependent conduit processes during magma ascent, such as crystallisation, gas exsolution and expansion, and fragmentation (Cassidy et al. 2018), and ranges from effusive lava flows to highly explosive activity, such as Plinian eruptions. Transitions in eruptive style have been related to the efficiency of outgassing, the process by which gas can decouple from the melt phase during magma ascent (Bamber et al. 2024; Bonechi et al. 2024a). Effusive eruptions occur when outgassing is efficient during ascent (Degruyter et al. 2012; La Spina et al. 2021). Conversely, if coupling between the gas and melt phases is maintained, magma eventually fragments, generating explosive eruptions (Degruyter et al. 2012). Insights into the processes which occur during magma ascent and their controls can be obtained through the investigation of textural properties such as vesicle size distributions, vesicle number densities and crystallinities (Blower et al. 2001; Shea et al. 2010; Moitra et al. 2013), as well as the properties that relate to the connected pore network, such as porosity, connected porosity, connectivity, tortuosity and the throat-pore size ratio (Polacci et al. 2008, 2009; Bouvet de Maisonneuve et al. 2009; Arzilli et al. 2016; Colombier et al. 2017, 2021; Baker et al. 2019; Valdivia et al. 2022; Bamber et al. 2024). In this regard, one of the most useful techniques is X-ray computed microtomography, through which we can directly visualise the volumetric pore network within the clast and characterise the 3D geometric properties of permeable pathways (Polacci et al. 2010). To date, several studies have investigated magma permeability and established complex porosity–permeability relationships which depend on pore network properties (Saar and Manga 1999; Rust and Cashman 2004; Degruyter et al. 2010; Polacci et al. 2014; Burgisser et al. 2017; Baker et al. 2019; Moitra and Houghton 2021). However, previous works focus on basaltic to rhyolitic compositions, whilst highly alkaline magmas, such as the basanitic and tephritic compositions that characterised the 2021 Tajogaite eruption (La Palma, Spain), have not yet been investigated. These magmas have a lower melt viscosity than corresponding calc-alkaline and tholeiitic compositions (Giordano et al. 2008), which could affect outgassing efficiency and consequently, the processes which occur during magma ascent within the conduit. In this study, we provide the first 3D dataset for pyroclasts from highly alkaline magma compositions. Here, we report a 3D visualisation and quantification of pore network parameters (e.g. vesicle size distributions, vesicle number densities, porosity, connected porosity, connectivity, tortuosity factor and the throat-pore size ratio) from tephra erupted during the 2021 Tajogaite eruption. Our sample suite includes lapilli clasts which span the entire duration of the eruption (from September to December), and are, therefore, associated with both a wide range in eruptive style i.e. lava fountains, Strombolian activity and ash-rich jets (Taddeucci et al. 2023), and a change in magma composition from tephritic to basanitic (Table 1). Our aim is to investigate the connection between variations in textural properties, pore network parameters and eruptive style over the duration of the 2021 Tajogaite eruption and for highly alkaline magma compositions in general.

**Table 1:** Sample information for the lapilli clasts analysed in this study

Sample	Eruption date	Stratigraphic unit	Eruptive activity	*MER (10^3^ kg s^−1^)	^§^Magma ascent rate (m s^−1^)	Magma composition	^χ^Phase assemblage
20 Sept	20-Sep-21	LU1.2 (Romero et al. 2022a)	Fountain	12	0.43	Tephrite	Cpx(10–30%) + Pl(1–20%) + Ox(1–8%) + Amp(1–2%) + Ol(1–2%)
22 Sept	22-Sep-21	LU1.4 (Romero et al. 2022a)	Fountain	3	0.21	Tephrite	Cpx(10–30%) + Pl(1–20%) + Ox(1–8%) + Amp(1–2%) + Ol(1–2%)
25 Sept	25-Sep-21	LU1.6 (Romero et al. 2022a)	Fountain	18	0.3	Tephrite	Cpx(10–30%) + Pl(1–20%) + Ox(1–8%) + Amp(1–2%) + Ol(1–2%)
26 Sept	26-Sep-21	LU2.1 (Romero et al. 2022a)	Strombolian	1.4	0.17	Tephrite	Cpx(10–30%) + Pl(1–20%) + Ox(1–8%) + Amp(1–2%) + Ol(1–2%)
16 Oct	16-Oct-21	MU2 (Bonadonna et al. 2022)	Strombolian	2.7 ± 0.6	-	Basanite	Cpx(10–30%) + Ol(10–15%) + Pl (2–15%) + Ox(1–4%)
15 Nov	15-Nov-21	MU6 (Bonadonna et al. 2022)	Ash-rich jet	2.7 ± 0.6	0.01–0.3	Basanite	Cpx(10–30%) + Ol(10–15%) + Pl (2–15%) + Ox(1–4%)
13 Dec	13-Dec-21	UU2 (Bonadonna et al. 2022)	Fountain	4.3 ± 0.8	-	Basanite	Cpx(10–30%) + Ol(10–15%) + Pl (2–15%) + Ox(1–4%)

^*^Mass eruption rate (MER) data for September samples are from Romero et al. (2022a), whilst data for October-December are from Bonadonna et al. (2022)

^§^Magma ascent rates for September samples are from Romero et al. (2022a), whilst those for November are from Bonechi et al. (2024b)

^χ^Phase abundance is from Pankhurst et al. (2022), Romero et al. (2022a, b), Ubide et al. (2023) and Bonechi et al. (2024b)

### Evolution in eruptive style and magma composition during the 2021 Tajogaite eruption

The 2021 Tajogaite eruption of Cumbre Vieja (La Palma, Canary Islands) occurred between 19 September and 13 December 2021, producing the most significant recent eruption in the Canary Islands in terms of volume (∼0.2 km^3^) and duration (85 days) (Day et al. 2022). The Tajogaite eruption was classified as a long-lasting, hybrid eruption due to the simultaneous occurrence of both lava flows and tephra from multiple vents. Most vents produced, contemporaneously, a variety of eruptive styles, such as effusive and explosive activity including lava flows, spattering, lava fountains, Strombolian activity and ash emission (Longpré and Felpeto 2021; Pankhurst et al. 2022; Bonadonna et al. 2022; Romero et al. 2022a; Taddeucci et al. 2023). The eruption was initially characterised by five days of intense explosive activity, consisting of alternating lava fountains and *rapid* Strombolian explosions (i.e. periods when there is an increased frequency of Strombolian explosions; Houghton et al. 2016) before the western flank of the edifice collapsed on 25 September. Between 21 and 24 September, long-lasting fountaining episodes (i.e. several minutes to hours) became more frequent, whilst simultaneous lava effusion and sporadic lava fountaining occurred from the N flank vent (Romero et al. 2022a). After the collapse, volcanic activity persisted with no significant change; however, a complete pause in the activity for about 10 h, concomitant with a drop in volcanic tremor, occurred on 27 September, after which Strombolian activity and lava fountaining resumed at the main vent. After 11 October, the activity changed to a phase of variable intensity, characterised by the interplay between ash-laden Strombolian activity and sporadic episodes of lava fountaining, whilst the opening of new vents produced ash columns with scarce juvenile material and abundant country rock lithics (Romero et al. 2022a). Lava effusion continued intermittently from the lowest vents or lateral, short-lived vents around the cone. The end of the eruption occurred on 13 December at 18:20 local time, when a strong ash emission produced a column 8 km in height, sustained almost continuously for over an hour (Romero et al. 2022a). The variability in eruptive style observed throughout the Tajogaite eruption has been interpreted as the result of a complex interaction between the gas emission rate, the fractionation of gas and magma between explosive and effusive vents, changes in conduit geometry and magma ascent rate (Bonadonna et al. 2022; Taddeucci et al. 2023; González-García et al. 2023; Burton et al. 2023; Bonechi et al. 2024b). The average mass eruption rate (MER; Table 1) is estimated as 3–4 × 10^3^ kg s^−1^ and remained relatively constant throughout the duration of the Tajogaite eruption (Bonadonna et al. 2022). Particularly, MER values range between 3–18 × 10^3^ kg s^−1^ before 25 September and ∼1.4 × 10^3^ kg s^−1^ for late September to early October (Romero et al. 2022a), whilst the MER is 2.7 ± 0.6 × 10^3^ kg s^−1^ for October and November (MU; Bonadonna et al. 2022) and 4.3 ± 0.8 × 10^3^ kg s^−1^ for December (UU; Bonadonna et al. 2022). These values suggest that at the beginning of the eruption there was a higher magma flux compared to the following weeks and months, in agreement with the observations of Taddeucci et al. (2023), which find that fountaining and comparatively high fluxes dominated during the activity in September, whilst ash emission and lower fluxes occurred in November. This variation is also reflected in the decrease in magma ascent rates (Table 1) from September (0.17–0.43 m s ^−1^; Romero et al. 2022a) to November (from 0.01 to 0.3 m s^−1^, for estimated melt H_2_O contents of 1 to 3 wt.%; Bonechi et al. 2024b), calculated using the microlite number density water exsolution rate meter (MND; Toramaru et al. 2008).

A sharp change in lava morphology (i.e. from voluminous and blocky to more fluid and rapid lavas; Ubide et al. 2023) occurred after the first eruptive break on 27 September, and was accompanied by a change in the mineralogical assemblage from amphibole-clinopyroxene-bearing tephrite to clinopyroxene-olivine-rich basanite (Day et al. 2022; Pankhurst et al. 2022; Romero et al. 2022a, b; Ubide et al. 2023; Bonechi et al. 2024b; Longpré et al. 2025; Table 1). Lava and tephra samples from the beginning of the eruption (September) are characterised by the presence of clinopyroxene, plagioclase, oxide, olivine and amphibole, whilst samples from the second part of the eruption (October–November) are characterised by clinopyroxene, olivine, plagioclase and oxide. Despite this variation in the phenocryst/microphenocryst assemblage, the groundmass of both the tephrite and basanite compositions has a similar microcrystalline assemblage, consisting of plagioclase, clinopyroxene, olivine, Fe–Ti oxides and glass. Recent studies (e.g.Day et al. 2022; Ubide et al. 2023) proposed that the beginning of the eruption (September) was driven by the recycling of a relatively evolved, hydrous crystal mush, which generated the amphibole-bearing tephritic magma. From the first eruptive break onward, there was a gradual increase in the more mafic basanitic magma component (Ubide et al. 2023; Longpré et al. 2025; Bonechi et al. 2024b), evidenced by the higher anorthite content in plagioclases from September (An_53–66_) to November (An_61–68_). This trend continued until the occurrence of a geochemical kink between 25 and 30 November, marking the transition from mafic recharge to melt fractionation (~ 10%; Ubide et al. 2023) and signalling the cessation of mantle magma supply (Longpré et al. 2025). Following this transition, an increase in incompatible element concentrations and a decrease in the mafic component of phenocryst rims and microcrysts (Ubide et al. 2023), as well as a reversal trend in tephra glass composition (Longpré et al. 2025), were recorded, marking the decline of mantle magma supply and foreshadowing the end of the eruption.

## Methods

### Samples

The samples used in this study are lapilli clasts (2–4 mm in diameter) collected during the 2021 Tajogaite eruption (La Palma, Spain). The sample suite consists of seven groups of lapilli clasts, belonging to the September (20 Sept, 22 Sept, 25 Sept, 26 Sept samples; Romero et al. 2022a), October (16 Oct sample), November (15 Nov sample; Bonechi et al. 2024b) and December (13 Dec sample) periods. The September samples are the same as those used in Romero et al. (2022a), where they were characterised in terms of grain size. The October–December samples belong to the same sample suite investigated in Bonadonna et al. (2022, 2023) and have also been characterised in terms of grain size.

All of the samples belong to the lower (LU), middle (MU) and upper units (UU) (Table 1) reported in the Bonadonna et al. (2022) and Romero et al. (2022a) stratigraphic columns. The samples were chosen to cover the entire eruption period (19 September–13 December) and the variation in both eruptive style (i.e. lava fountain, Strombolian activity, ash-rich jet) and magma composition between September–early October (tephritic) and late October–December (basanitic). The eruptive styles associated with the investigated samples were assigned based on eyewitness observations and qualitative field descriptions (two of the authors, JER and MB, were in the field during the activity) made during the eruption, focusing on the predominant behaviours at different vents. These descriptions are in agreement with findings from other studies (e.g. Carracedo et al. 2022; Bonadonna et al. 2022, 2023; Romero et al. 2022a, b; Taddeucci et al., 2023), ensuring consistency with previously documented eruptive processes.

The LU samples were collected from a stratigraphic sequence located 1.3 km northeast of the active vents, at the Llano del Jable Astronomical viewpoint. At this locality, the sequence was 19.5 cm thick by the time of description (5 October 2021). The chronology of tephra deposition, for each tephra layer, was determined through detailed in situ juvenile tephra fall out observations (Bonadonna et al. 2022) and the textural characterisation of juvenile fragments in ash trays as described in Rodríguez et al. (2024) framed in a collective reconstruction of the eruption products under the umbrella of INVOLCAN. For the lower units (LU1.1–2.1), the proportion of lithic clasts is lower than 20%, and the juvenile material dominated by sideromelane and tachylite. These scoria fragments have poorly sorted, fine-to-very-fine skewed unimodal distributions and contained coarse ash to medium-to-coarse lapilli (Romero et al. 2022a), in which the largest clasts collected ranged from 1.5 to 2.0 cm in diameter (Bonadonna et al. 2022). In comparison, samples MU2, MU6 and UU2 were all sampled from fresh surfaces during in situ deposition. The first two samples were collected ~ 3.2 km north-northwest of the fissure in Tacande, whereas the latter was collected in Tajuya, ~ 3.5 km northwest of the fissure. They all correspond to fine juvenile particles to medium lapilli. The size of the particles sampled lie within > 45% mass weight of their corresponding tephra deposit distribution.

### Synchrotron based X-ray computed microtomography acquisition

The X-ray microtomographic acquisitions were performed at the I12-JEEP beamline (Diamond Light Source, United Kingdom), using propagation to enhance the phase contrast with a sample-detector distance of 2200 mm, and a monochromatic beam (53 keV). For each scan, 1800 tomographic projections were acquired by the detector with equiangular steps (2.174 deg s^−1^), over a full rotation angle of 180°. The exposure time for each projection was 0.046 s, thus, the acquisition time of each scan was ~ 83 s. The isotropic pixel size was 3.24 μm. The detector is a high-resolution imaging PCO edge camera with optical module 3, corresponding to a field of view of 8.0 × 7.0 mm. The resulting tomographic projections were reconstructed into 2D slices using the Savu program (Wadeson and Basham 2016) (https://savu.readthedocs.io/en/latest/tutorials/confluence/I12/SAVU-Tomography-Reconstruction.html). The pre-processing pipeline includes the centre of rotation calculation (Vo et al. 2014), zinger removal, blob removal (Vo et al. 2018) and regularisation-based ring removal (Vo et al. 2018).

### Synchrotron-based X-ray computed microtomography: image processing and analysis

Here we provide further details on the image processing procedure and quantitative textural analysis. Samples were not cut into smaller volumes before acquisition, to preserve the original morphology of the lapilli clasts. For almost all the samples, two to four separate, but overlapping sub-volumes were extracted from the same volume acquired originally (corresponding to sub-volumes smaller than ~ 3 mm^3^) during data processing and analysis. Further details on the pre-segmentation and segmentation image processing procedure and quantitative image analysis are provided in Online Resource 1.

#### Pre-segmentation and segmentation image processing

First, we tested several sub-volumes in order to constrain the representative elementary volume (REV; Bear 1972) for the entire workflow. For a better visualization of the obtained data, this study focuses on the data obtained from the REV (Tables 1 and 2). However, for completeness, the pore network parameters obtained for all sub-volumes are reported in Table S1 in Online Resource 2. Following the stacking of images to produce complete sample sub-volumes in ImageJ (Abramoff et al. 2004; Schneider et al. 2012), the image processing and analysis was completed using the commercial software Avizo (v. 2019.1; Thermo Fisher Scientific, USA). The segmentation of vesicle and crystal volumes from the surrounding matrix glass was operated in the 3D domain by using manual bi-level greyscale thresholding, based on the greyscale histogram of the selected VOIs and visual inspection of the slices in different directions (axial, coronal and sagittal). Orthoslices of the 3D reconstructed volumes (Fig. S1 in Online Resource 3) were then compared with 2D backscattered electron (BSE) images (Fig. S1 in Online Resource 3) to examine vesicle morphologies and to further validate the 3D image processing procedure.

**Table 2:** Resu﻿lts of the 3D quantitative textural analysis on representative elementary volumes reconstructed from synchrotron-based X-ray microtomographic images

Sample	Eruption date	Eruptive activity	Connected porosity (*Φ*_c_)	Connectivity	Volume tot vesicles (μm^3^)	Volume sample (μm^3^)	Porosity (*Φ*)	Bubble diameter (μm)	σ	Bubble diameter (m)
20 Sept_a	20-Sep-21	Fountain	0.646	0.999	8.9 × 10^8^	1.4 × 10^9^	0.65	80	48	8 × 10^–5^
22 Sept_a	22-Sep-21	Fountain	0.610	0.999	1.5 × 10^9^	2.4 × 10^9^	0.61	85	47	9 × 10^–5^
25 Sept_a	25-Sep-21	Fountain	0.538	0.998	7.5 × 10^8^	1.4 × 10^9^	0.54	83	43	8 × 10^–5^
26 Sept_a	26-Sep-21	Strombolian	0.319	0.956	8.3 × 10^8^	2.5 × 10^9^	0.33	67	47	7 × 10^–5^
16 Oct_a	16-Oct-21	Strombolian	0.389	0.973	6.1 × 10^8^	1.5 × 10^9^	0.40	71	39	7 × 10^–5^
15 Nov_a	15-Nov-21	Ash-rich jet	0.552	0.986	1.4 × 10^9^	2.5 × 10^9^	0.56	62	48	6 × 10^–5^
13 Dec_a	13-Dec-21	Fountain	0.597	0.999	2.1 × 10^9^	3.5 × 10^9^	0.60	92	62	6 × 10^–5^
Sample	^§^VND (mm^−3^)	^χ^VND_m_ (mm^−3^)	Log10 VND_m_	*Crystal volume fraction (*φ*_c_)	Tortuosity (*τ*)	σ	Tortuosity factor (*m*)	σ	Throat-pore size ratio (*f*_*tb*_)	*σ*
20 Sept_a	9.6 × 10^2^	2.6 × 10^3^	12.41	0.42	1.54	0.46	2.78	1.25	0.32	0.17
22 Sept_a	7.8 × 10^2^	1.8 × 10^3^	12.27	0.60	1.55	0.45	2.61	1.11	0.34	0.18
25 Sept_a	9.0 × 10^2^	1.9 × 10^3^	12.27	0.52	1.49	0.46	2.16	0.89	0.36	0.18
26 Sept_a	6.5 × 10^2^	9.7 × 10^2^	11.99	0.62	1.48	0.48	1.61	0.50	0.34	0.19
16 Oct_a	1.0 × 10^3^	1.7 × 10^3^	12.22	0.55	1.49	0.48	1.76	0.60	0.35	0.17
15 Nov_a	1.0 × 10^3^	2.3 × 10^3^	12.35	0.47	1.58	0.57	2.37	1.04	0.29	0.16
13 Dec_a	4.7 × 10^2^	1.1 × 10^3^	12.06	0.57	1.56	0.65	2.50	1.26	0.32	0.18

^*^φ_c_ = Crystal volume fraction (calculated on vesicle free basis). It refers to phenocrysts and microphenocrysts; microlites too small to be analysed

^§^VND represents the bubble number density calculated as the number of vesicles per m^3^ of the total sample volume

^χ^VND_m_ represents the bubble number density calculated as the number of vesicles per mm^3^ of the total melt volume

The bubble diameter, tortuosity (*τ*), tortuosity factor (*m*) and throat-pore size ratio (*f*_*tb*_) are reported as mean; σ is the standard deviation

#### Quantitative image analysis

Porosity and connected porosity were calculated using algorithms available in Avizo, following the approach of Bamber et al. (2024). Both the porosity (*Φ*) and connected porosity (*Φ*_c_) were calculated with respect to the total sample volume. The errors for porosity and connected porosity were assessed by comparing the difference in the calculated values using two different thresholds for the segmentation of vesicles for the sample volume, with one threshold selected on the basis of preservation of the thin melt films separating vesicles and the second one chosen for greater segmentation of vesicles. The average error for porosity was ± 0.02 and the average error for connected porosity was ± 0.06. The crystal volume fraction (*φ*_c_) was obtained by dividing the total volume of crystals by the total vesicle-free sample volume, which consists of only the volume of glass and crystals.

To calculate vesicle number densities (VND) and size distributions (VSD), the connected vesicles needed to be separated. The *separate objects* algorithm in Avizo was used for this purpose, which separates objects using a marker-based watershed algorithm, combining a Chamfer distance map and H-maxima. The algorithm computes the watershed lines of a binary image. A marker extent of 1 was deemed most suitable for separating the smallest vesicles, whilst also preserving the volume of larger vesicles (see Online Resource 1). The VND was calculated as the number of vesicles per m^3^ of the total sample volume (Table 2). The VND_m_, instead, was calculated as the number of vesicles per m^3^ of the total melt volume (Table 2).

To calculate sample tortuosity (*τ*) i.e. the ratio between the actual length of the flow path and the length of the shortest possible path (a straight line) in the direction of flow (represented by a value of 1), a skeleton was constructed for each sample using the *auto skeleton* algorithm in Avizo. The data obtained were processed using the MATLAB script of Bamber et al. (2024), to calculate the tortuosity for each segment connecting three nodes. The tortuosity was used to calculate the tortuosity factor (*m*), following Degruyter et al. (2010):

1 $${\tau }^{2}= {\Phi }_{\text{c}}^{1-\text{m}}$$

Both *τ* and *m* were computed for each set of segments. The mean and standard deviation (1σ) of *τ* and *m* are reported for each sample in Table 2.

To calculate the throat-pore size ratio (*f*_*tb*_) i.e. the ratio between the radius of the throat and vesicle (calculated as an average of the radii of the two connected vesicles), the *generate pore network model* algorithm was used in Avizo. The output data (i.e. radii of connected pores and throats) were incorporated in the MATLAB script of Bamber et al. (2024) to calculate the throat-pore size ratio as follows:

2 $${f}_{tb}= \frac{{t}_{r}}{{mean}_{r}}$$

where the *mean*_*r*_ represents the average radii of the two connected pores, and *t*_*r*_ represents the radius of the throat which connects them. The *f*_*tb*_ is reported as a mean and standard deviation (1σ) for each sample in Table 2.

### Scanning electron microscopy

BSE images were collected using a JEOL JSM-6390LA FE-SEM at the Department of Earth and Environmental Sciences, University of Manchester, United Kingdom, to analyse vesicle shapes and crystal morphologies. We used an acceleration voltage of 15 kV and a working distance of 10 mm.

## Results

### Vesicle shapes, size distributions and number densities in tephra clasts

**Fig. 1:**
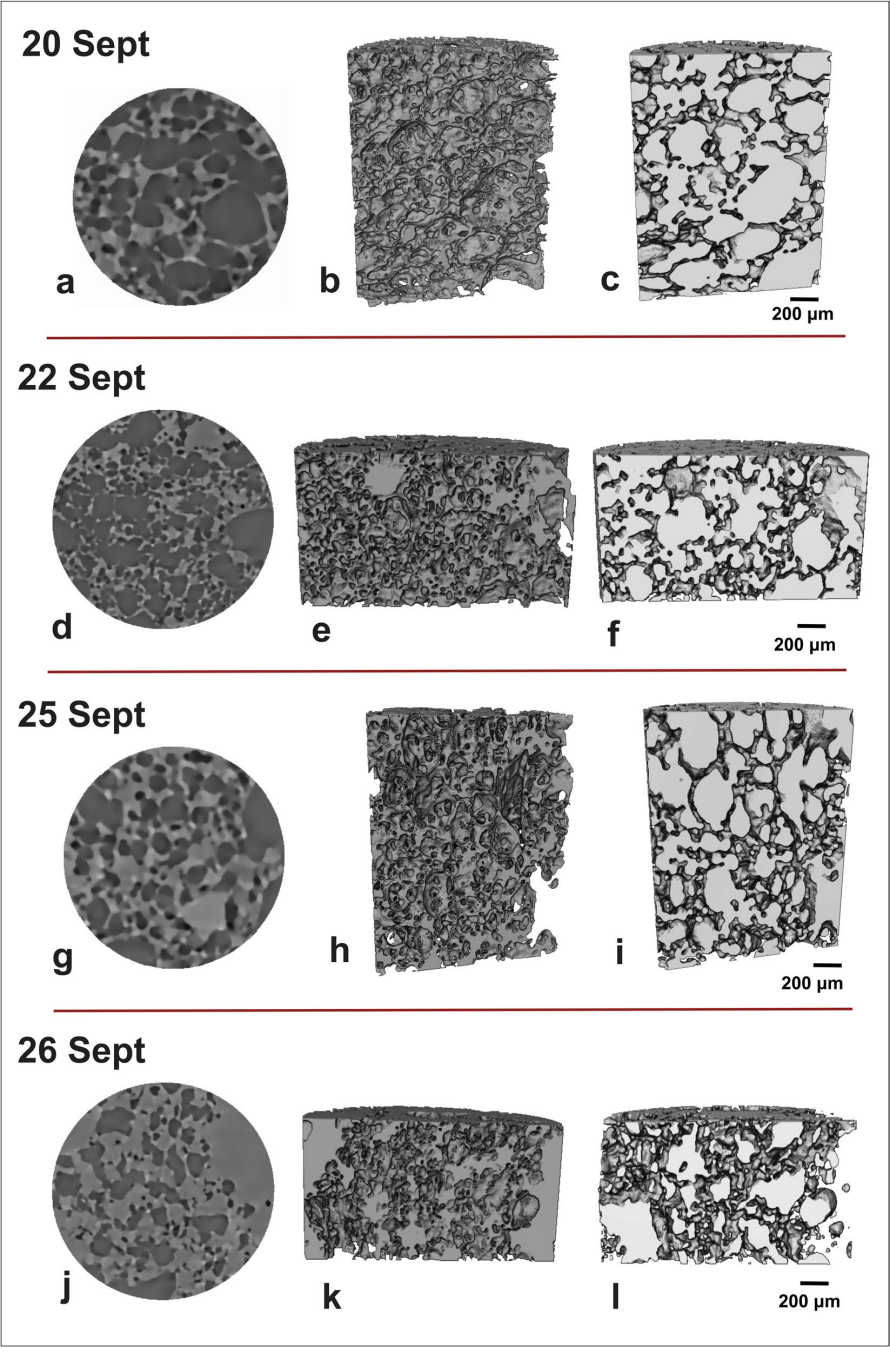
Representative 3D volume renderings of September tephra samples: **a**–**c** 20 Sept, **d**–**f** 22 Sept, **g**–**i** 25 Sept and **j**–**l** 26 Sept. Panels **b**, **e**, **h** and **k** show renderings (voxel size = 3.24 μm), which focus on the matrix glass and crystals, whilst panels **c**, **f**, **i** and **l** show renderings which focus on the segmented vesicles. All sample volumes are ≤ 3 mm^3^. The height and the diameter of the cylinder for the 20 Sept and 25 Sept samples are 1.3 mm and 0.9 mm; for the 22 Sept and 26 Sept samples, instead, it is 1 mm and 1.7 mm. Volume renderings were produced using the Avizo software

**Fig. 2:**
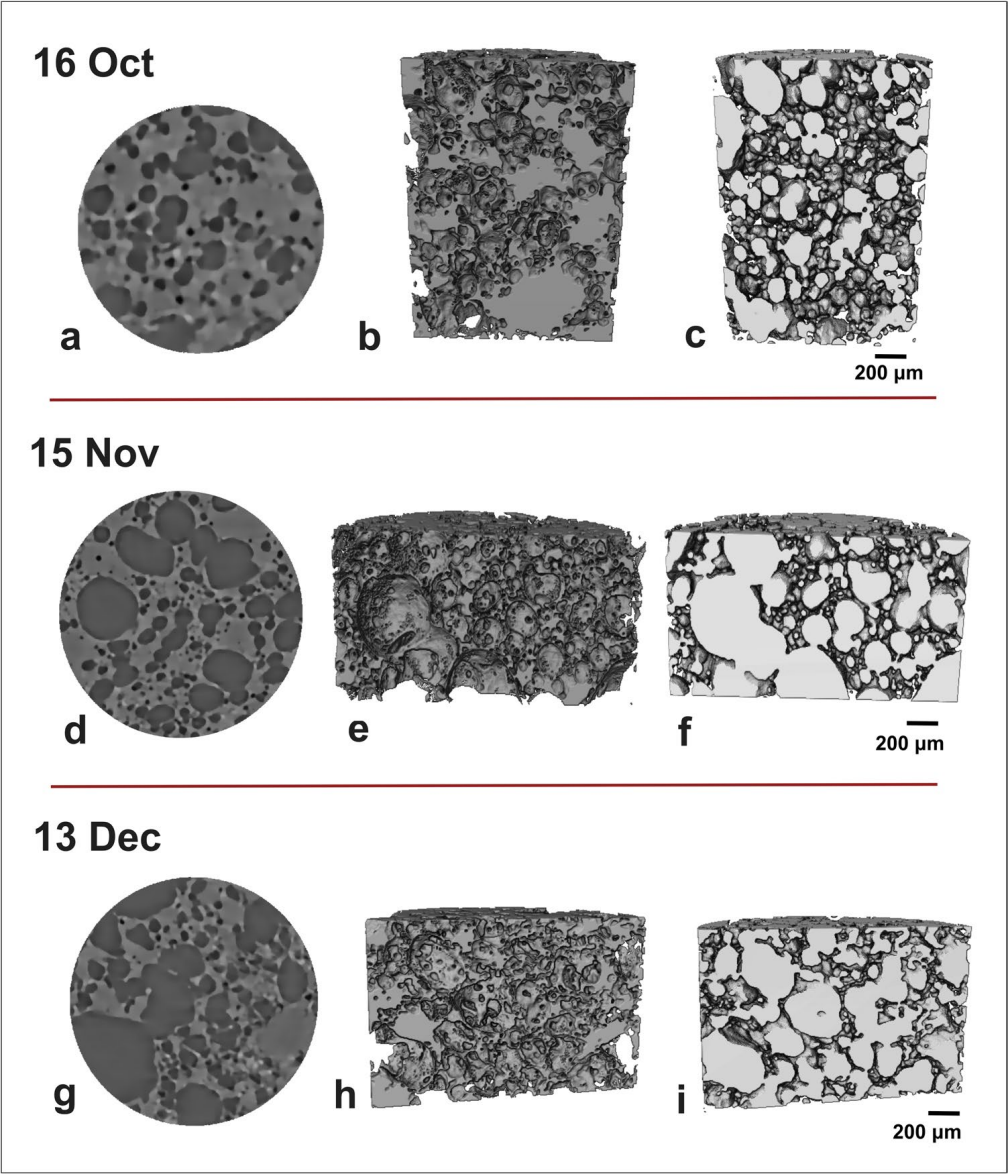
Representative 3D volume renderings of October, November and December tephra samples: **a**–**c** 16 Oct, **d**–**f** 15 Nov and **g**–**i** 13 Dec samples. Panels **b**, **e** and **h** show renderings (voxel size = 3.24 μm), which focus on the matrix glass and crystals, whilst panels **c**, **f** and **i** show renderings which focus on the segmented vesicles. All sample volumes are ≤ 3 mm^3^. The height and the diameter of the cylinder for the 16 Oct sample are 1.3 mm and 0.9 mm; for the 15 Nov and 13 Dec samples, it is 1 mm and 1.7 mm. Volume renderings were produced using the Avizo software

The images and digital volumes obtained via synchrotron X-ray microtomography allow visualization and quantification of the vesicle textures of the Tajogaite lapilli clasts in 3D (Figs. 1 and 2; Online Resource 2). Amongst the September lapilli clasts, the 20 Sept, 22 Sept and 25 Sept samples show a high number of ellipsoidal to polylobate vesicles (Fig. 1), whilst for the 26 Sept sample, the vesicle volume fraction is lower and vesicles have a more irregular shape. Instead, October and November samples, in general, show mostly spherical to subspherical vesicles with a regular shape (Fig. 2).

**Fig. 3:**
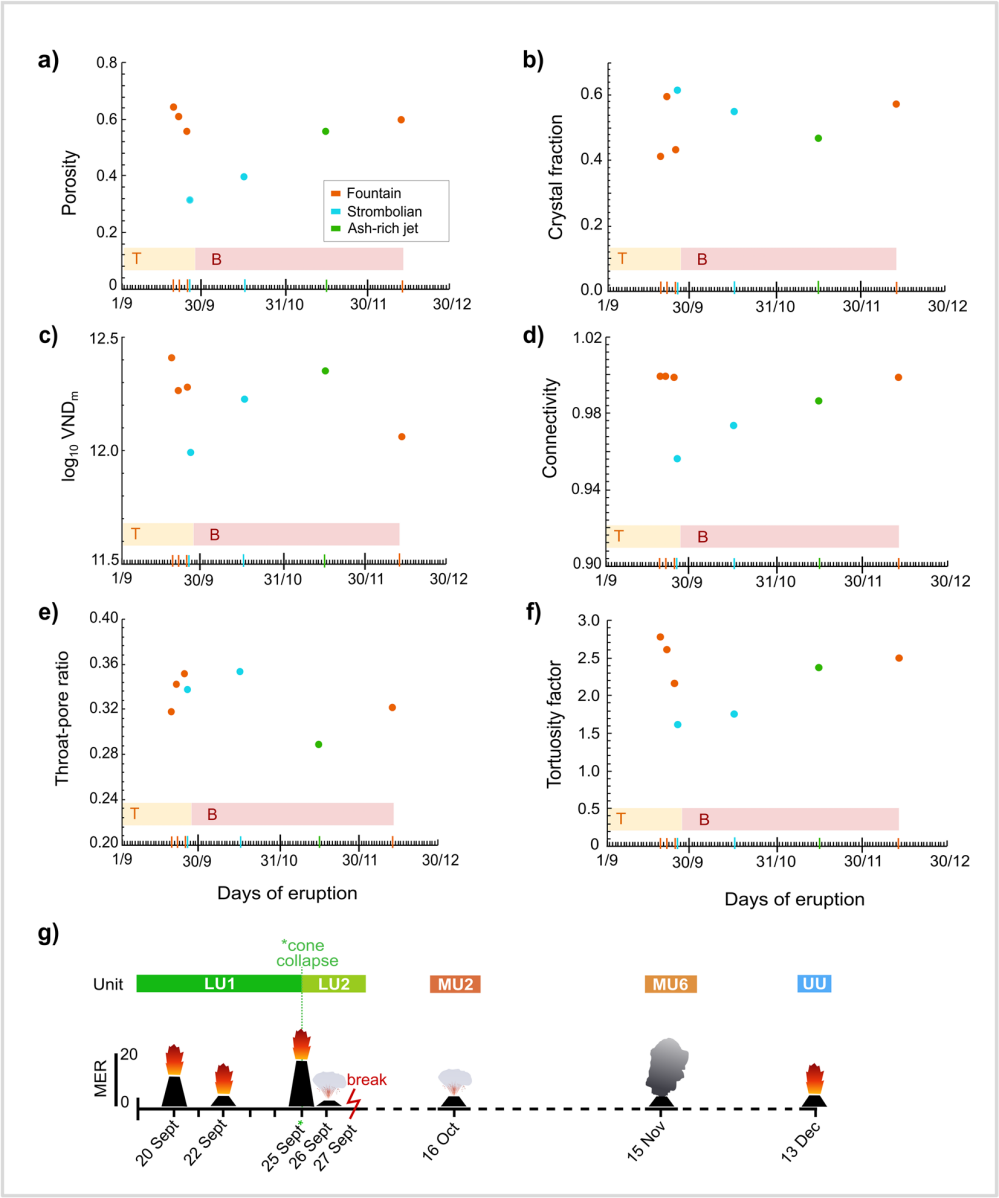
Diagrams showing the variation in **a** porosity, **b** crystal fraction, **c** log_10_VND_m_, **d** connectivity, **e** the throat-pore size ratio and **f** the tortuosity factor over the duration of the eruption. Also shown is a **g** diagram illustrating the evolution of the Tajogaite 2021 eruption and variation in eruptive intensity. Cone height indicates mass eruption rate (MER; Table 1). Units are from Bonadonna et al. (2022) and Romero et al. (2022a). The coloured boxes indicate the change in magma composition from tephritic (T) to basanitic (B)

Samples show low (~ 0.3) to moderate (~ 0.6) porosity (*Φ*; Fig. 3a and Table 2), and moderate to high crystallinity (*φ*_c_ = ~ 0.4–0.6; Fig. 3b and Table 2). Similar high crystallinity values have also been observed for samples of other basaltic lava fountain and Strombolian eruptions (Polacci et al. 2006, 2009; Romero et al. 2022b). Particularly, the 26 Sept (*Φ* = 0.33; *φ*_c_ = 0.62) and 16 Oct (*Φ* = 0.40; *φ*_c_ = 0.55) samples show lower porosity and higher crystal fraction values than those of the 20 Sept (*Φ* = 0.65; *φ*_c_ = 0.42), 25 Sept (*Φ* = 0.56; *φ*_c_ = 0.44) and 15 Nov (*Φ* = 0.56; *φ*_c_ = 0.47) samples. The 22 Sept (*Φ* = 0.61; *φ*_c_ = 0.60) and 13 Dec (*Φ* = 0.60; *φ*_c_ = 0.57) samples, instead, show both high porosity and high crystal fraction values (Table 2). As shown in Fig. 3a, porosity varies over the duration of the eruption. Indeed, for clasts erupted at the beginning of the eruption, there is a sharp decrease in porosity (from 0.65 to 0.33) following the 25 September collapse, after which the porosity increases, reaching pre-collapse values towards the end of the eruption.

**Fig. 4:**
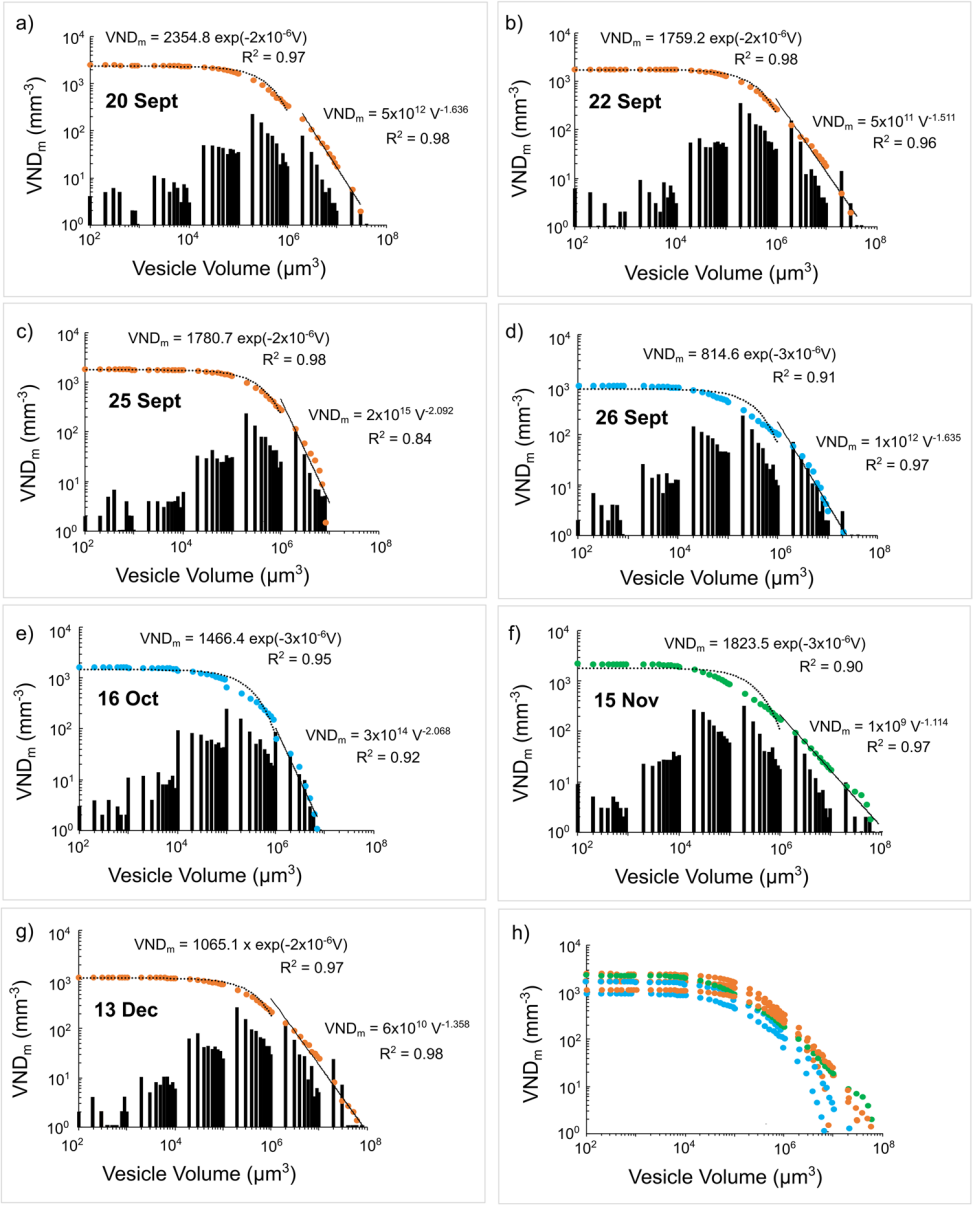
**a**–**g** Cumulative (circles) and noncumulative (bars) vesicle size distributions for REV tephra samples of September (20 Sept, 22 Sept, 25 Sept, 26 Sept), October (16 Oct), November (15 Nov) and December (13 Dec). The VND_m_ represents the bubble number density calculated as the number of vesicles per mm^3^ of the total melt volume. The solid black lines show where vesicle volume distributions can be described by a power-law trend, whilst the dashed black curves show where vesicle size distributions can be described by an exponential trend. All vesicle size distributions have mixed power-law-exponential distributions. **h** Cumulative (circles) vesicle size distributions for all REV tephra samples

The vesicle size distributions of our lapilli clasts are expressed as the number of vesicles per mm^3^ within each volume class, and are shown as both cumulative and frequency distributions on a log–log plot (Fig. 4 and Online Resource 4). The cumulative distribution, or a portion of it, can typically be fit by mathematical relations (i.e. exponential, power law, etc.), where the exponent provides insight into the vesiculation processes occurring in magmas (Gaonac’h et al. 1996; Blower et al. 2001; Bai et al. 2008). In all samples, individual vesicle volumes span approximately 6–7 orders of magnitude, with values ranging between 3 × 10^1^ μm^3^ and a maximum of ~ 6 × 10^7^ and 2 × 10^8^ μm^3^ (Online Resource 4). For all lapilli clasts, vesicle size distributions (VSDs) can be described by a power law trend when vesicle volumes exceed 10^6^ μm^3^ with an exponent between 1.1 and 2.1 (Fig. 4; Online Resource 4). Instead, we note that VSDs for volumes between 10^1^ and 10^6^ μm^3^ can be described by an exponential trend (Fig. 4; Online Resource 4). Vesicle number densities (VND_m_) vary between ~ 9 × 10^2^ mm^−3^ and ~ 2 × 10^3^ mm^−3^ (log_10_ VND_m_ from 11.9 to 12.4; Table 2 and Table S1 in Online Resource 2), with the maximum value measured in the 20 Sept sample (Fig. 3c).

### Connectivity, throat-pore size ratio and tortuosity factor

The 3D geometry of the pore network, such as the connectivity, throat-pore size ratio and tortuosity of connections, strongly affects magma permeability, and, in turn, outgassing efficiency and explosivity (Colombier et al. 2021; Bamber et al. 2024). Vesicle connectivity (C) was calculated as the ratio of connected porosity over porosity, following Colombier et al. (2021):

3 $$\text{C}={\Phi }_{\text{c}}/\Phi$$

**Fig. 5:**
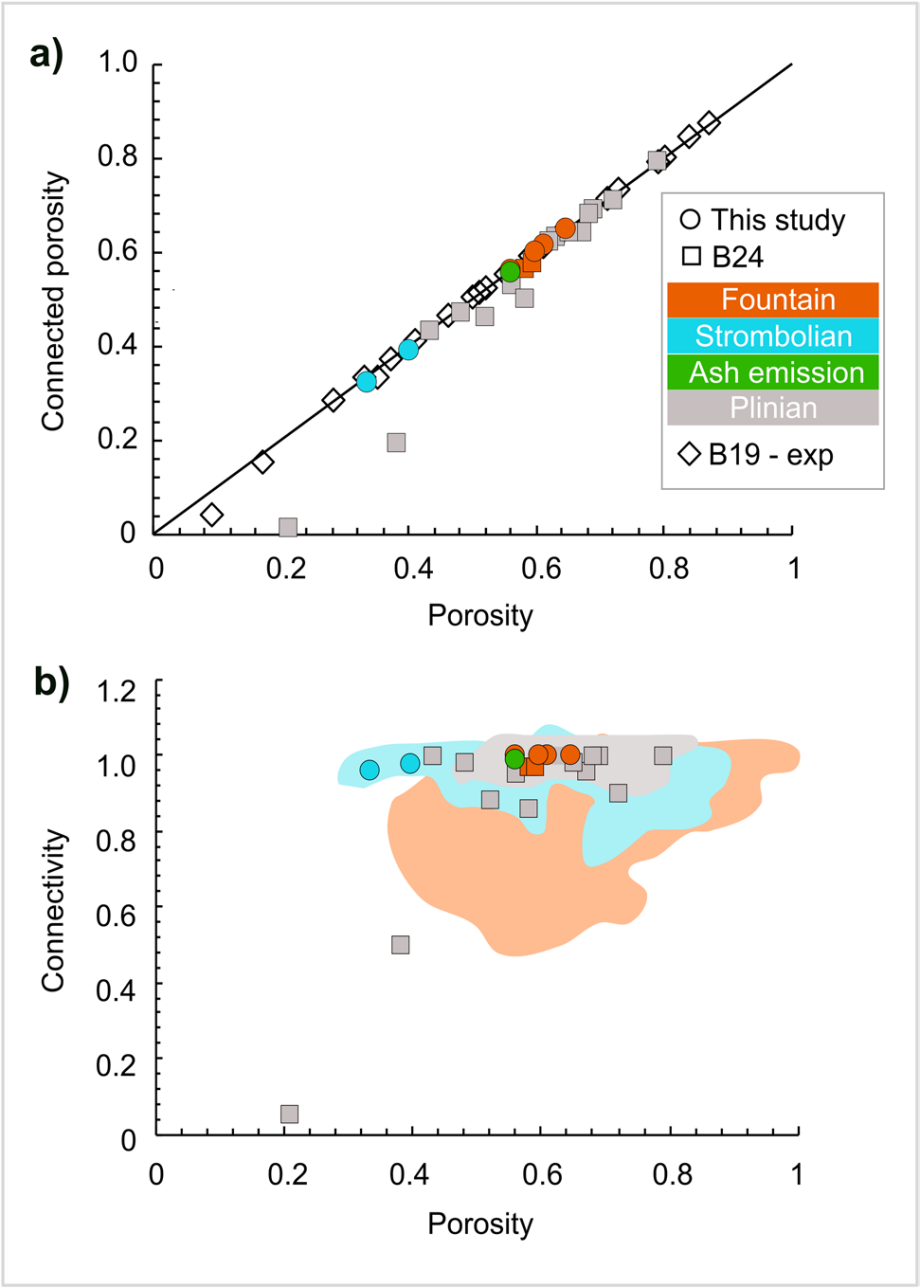
Diagrams showing **a** connected porosity and **b** connectivity vs porosity for our dataset. Literature data as follows: B19 = Baker et al. (2019); B24 = Bamber et al. (2024). For Fig. 5b, coloured fields are data from Colombier et al. (2021). The coloured fields represent fountain (orange), Strombolian (light blue), ash-rich jet (green) and Plinian (grey) activity. B19 is not classified into eruption style. The black line in Fig. 5a represents a connectivity of 1

Overall, connectivity is high, ranging between 0.96 and 0.99 (Table 2; Figs. 3d and 5a,b).

Generally, the samples with high values of porosity (*Φ* = 0.6) show the highest connectivity (C = 0.99), whilst those with the lowest porosity (*Φ* = 0.3–0.4) show the lowest connectivity (C = 0.96–0.97) (Fig. 5b). The average vesicle size (equivalent diameter, Ø; Table 2) is higher (Ø = 80–85 μm) for samples with higher connectivity (20 Sept, 22 Sept, 25 Sept, 13 Dec), and lower (Ø = 67 μm) for samples with lower connectivity (26 Sept, 16 Oct, 15 Nov).

The throat-pore size ratio shows a narrow range (0.29–0.35) across all of the lapilli clasts of the Tajogaite eruption (Fig. 3e and Table 2).

The tortuosity factor calculated for the September lapilli clasts varies between 1.61 and 2.78, whilst the tortuosity factor for lapilli clasts from October, November and December is 1.76, 2.37 and 2.5 respectively (Table 2). The tortuosity factor shows a trend that is similar to that observed for porosity (Fig. 3f). Particularly, the 26 Sept and 16 Oct samples show a lower tortuosity factor compared to the 20 Sept, 25 Sept and 15 Nov samples. As shown in Fig. 3f, the tortuosity factor varies throughout the eruption. Notably, for clasts erupted at the beginning of the eruption there is a sharp decrease in the tortuosity factor (from 2.78 to 1.61). Following the collapse event on 25 September, instead, the tortuosity factor increases again, reaching pre-collapse values towards the end of the eruption.

## Discussion

### Variation in pore network properties and the link with eruptive style during the 2021 Tajogaite eruption

The variations in texture and pore network parameters observed in our samples may be related to the changes in eruptive style which were observed over the course of the Tajogaite eruption, from lava fountain to Strombolian and ash-rich jet activity. However, we recognise that as we are examining the erupted products, post-fragmentation processes such as continued vesiculation, bubble shrinkage and densification, may also have potentially modified primary textures following ejection of the clasts from the vent (Stovall et al. 2011; Colombier et al. 2021).

The samples which belong to Strombolian and ash-rich jet activity (26 Sept, 16 Oct and 15 Nov) overall show more regular shaped vesicles (Fig. 2) than those of September and December which were erupted from lava fountain activity. Noteworthy, the presence of vesicles with a more irregular shape in the 26 Sept sample may be related to the higher groundmass crystallinity (*φ*_c_ = 0.62; Table 2), as bubbles deform around the rigid crystalline network (Sable et al. 2006; Costantini et al. 2010; Bamber et al. 2020, 2024). We also observed a similar trend for the average dimension of the vesicles (expressed as equivalent diameter) (Table 2). Samples from lava fountain activity show the highest equivalent diameter, whilst those belonging to Strombolian and ash-rich jet activity show lower values. Amongst the other network parameters investigated, the VND_m_ and *f*_*tb*_ also appear to vary with eruptive style. For the September samples, the observed VND_m_ tends to increase with eruption intensity (Sable et al. 2009; Potter et al. 2019; Bamber et al. 2024). Indeed, higher VNDs may reflect fewer connections between vesicles, favouring an increase in gas–melt coupling and the probability of magma fragmentation (La Spina et al. 2017). Similarly, the low values of *f*_*tb*_ calculated for the 15 Nov sample suggest that magma permeability was low and gas–melt coupling was maintained for the more explosive, ash-rich jet. Indeed, lower values of *f*_*tb*_ decrease magma permeability, increasing the likelihood of an explosive eruption (Degruyter et al. 2012; La Spina et al. 2017). However, since the *f*_*tb*_ values calculated for the other samples of the eruption are not considerably higher, for this eruption, the *f*_*tb*_ may have had less of a control on explosivity compared to other parameters such as the VND, also observed when comparing clasts of basaltic activity (Bamber et al. 2024).

The differences in vesicle shape, porosity, connectivity and, to a lesser extent, the throat-pore-size ratio across our sample suite may reflect a change in outgassing conditions during the Tajogaite eruption (Fig. 3). In the case of Strombolian activity, the lower VND_m_ and *m* observed in the clasts suggests that there are fewer vesicles and the channels which connect them are less tortuous than for clasts from fountain and ash-rich jet activity, favouring a lower degree of gas–melt coupling and thus, more efficient outgassing. In the case of lava fountain and ash-rich jet activity, instead, the higher VND_m_ and *m* suggest that there are a higher number of vesicles, connected by slightly tortuous channels, promoting some degree of gas–melt coupling and thus, less efficient outgassing. Furthermore, for ash-rich jets, the presence of narrower channels, as suggested by the lower *f*_*tb*_, favours a greater degree of gas–melt coupling with respect to fountain activity, leading to magma fragmentation.

Magma ascent rate also has considerable influence on outgassing efficiency (Bamber et al. 2024). Low magma ascent rates promote bubble coalescence and allow gas–melt decoupling, increasing the likelihood of a Strombolian or effusive eruption (Parfitt and Wilson 1995; Polacci et al. 2008). Instead, high magma ascent rates likely limit the time available for gas–melt decoupling during ascent, leading to magma fragmentation and a highly explosive eruption if magma viscosity is high (Szramek 2016; Arzilli et al. 2019). For low-viscosity magmas where high magma ascent rates may restrict gas–melt decoupling, high-intensity fountain activity may result (La Spina et al. 2021). The average magma ascent rates estimated for the 2021 Tajogaite eruption (Romero et al. 2022a; Bonechi et al. 2024b; Table 1) are higher for fountain activity on the 20 and 25 September (0.43 and 0.30 m s^−1^, respectively) than for other activity during September (~ 0.20 m s^−1^). According to Romero et al. (2022a), the faster ascent velocities estimated for activity on the 20 and 25 September reflect magma acceleration and fragmentation outside the vent (e.g. Cimarelli et al. 2010; La Spina et al. 2021), and lower magma viscosities before the cone collapse. Instead, the lowest ascent velocities estimated for the 26 September are coherent with the 10-h pause in eruptive activity which resumed on 27 September. Regarding the activity on 15 November, the estimated ascent velocities show a wider range of values (~ 0.01–0.3 m s^−1^; Table 1) that is up to 1 order of magnitude lower than those estimated for the activity of September. It is important to highlight that the range in estimates for the ascent rate reflects the range in water content estimated for the melt (1 to 3 wt.%; Bonechi et al. 2024b). These velocities, overall, are coherent with magma acceleration and fragmentation in the shallower part of the conduit, as suggested by the similarity of their values with those of sustained flows in eruptive conduits, and by the rapid (order of minutes) crystallisation of plagioclase microlites (Bonechi et al. 2024b). Indeed, rapid syn-eruptive crystallisation during magma ascent within the conduit can be driven by degassing and cooling during conditions of high undercooling, inducing a step change in viscosity which can trigger magma fragmentation within the conduit (La Spina et al. 2015; Arzilli et al. 2019; Bamber et al. 2022). Overall, the ascent rates estimated for the activity recorded during the Tajogaite eruption (Table 1) are in agreement with the development of an intense hybrid eruption, with similar estimated ascent rates to the 2014–2015 Holuhraun eruption (Iceland), which range between 0.12 and 0.29 m s^−1^ (Hartley et al. 2018).

### Comparison with other eruptive styles

To investigate the influence of the pore network parameters on eruptive style, we compare our dataset characterised by fountains, ash-rich jets and Strombolian activity with literature data spanning from fountain to Plinian activity (Degruyter et al. 2010; Polacci et al. 2008, 2009, 2012; Colombier et al. 2021; Valdivia et al. 2022; Bamber et al. 2024).

Mixed power-law exponential VSDs were observed in all samples of the Tajogaite eruption (Fig. 4). VSD power-law fits indicate continuous, multiple vesicle nucleation events and coalescence due to non-equilibrium degassing behaviour (Blower et al. 2001, 2003; Bai et al. 2008; Polacci et al. 2009; Baker et al. 2012; Le Gall and Pichavant 2016). A shift from a power law to exponential VSD indicates a transition towards equilibrium degassing, where vesicles are of similar size and separated by comparable distance (Bai et al. 2008; Polacci et al. 2009; Baker et al. 2012; Le Gall and Pichavant 2016). The VSDs of the samples investigated in this study suggest that bubble growth and coalescence may be superimposed upon a continuous bubble nucleation mechanism, reflecting an evolution towards equilibrium conditions.

To further evaluate vesiculation dynamics, we compared our results with 3D textural studies of basaltic scoria clasts from activity at Stromboli (i.e. mild to moderate intermittent explosions; Polacci et al. 2008, 2009) and Ambrym (i.e. lava fountaining generating a fallout deposit; Polacci et al. 2012) volcanoes. These two datasets provide an appropriate reference, as other vesicle studies of basaltic deposits (e.g. Mangan and Cashman 1996; Mastin et al. 2004; Sable et al. 2006, 2009; Lautze and Houghton 2007; Gurioli et al. 2008, 2014, 2018; Stovall et al. 2011, 2012; Parcheta et al. 2013) were conducted in 2D and are not directly comparable with 3D measurements. The VNDs of Tajogaite lapilli (~ 4 × 10^2^ to ~ 1 × 10^3^ mm⁻^3^) fall within the range reported for Stromboli (5 × 10^2^ to 2 × 10^3^ mm⁻^3^) and Ambrym (0.2 × 10^3^ to 1.7 × 10^3^ mm⁻^3^) scoria clasts, suggesting similarities in vesicle nucleation and growth processes. The VSDs of the Tajogaite lapilli span a wide range of vesicle volumes (~ 3 × 10^1^ to ~ 2 × 10⁸ μm^3^) and exhibit both power-law and exponential trends, comparable to observations from the Stromboli and Ambrym scoria clasts. The similarities in VND and VSD trends comparing the Tajogaite lapilli and Stromboli and Ambrym scoria clasts suggest that both systems underwent comparable vesiculation dynamics.

**Fig. 6:**
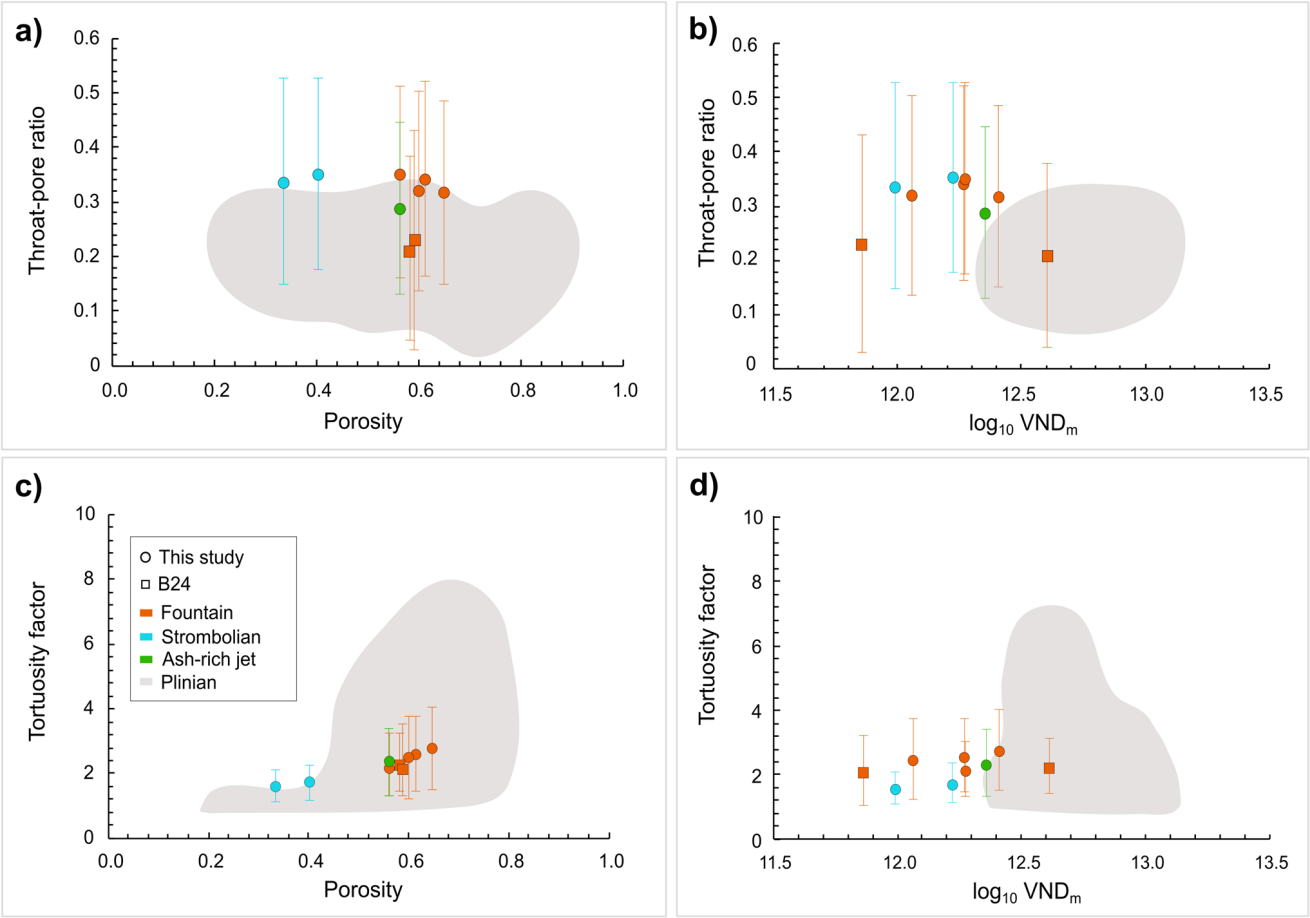
Diagrams showing the **a** throat-pore size ratio and the **c** tortuosity factor vs porosity and the **b** throat-pore size ratio and the **d** tortuosity factor vs log_10_VND_m_. Legend as Fig. 5. Literature data are as follows: B24 = Bamber et al. (2024). Plinian data are from Valdivia et al. (2022) and Bamber et al. (2024)

The Tajogaite samples have values of *m* (1.6–2.8; Table 2) comparable with those estimated for the basaltic fountain events at Etna (Fig. 6c,d; orange squares) (Bamber et al. 2024), and lower than estimated values of *m* for basaltic Plinian samples (Valdivia et al. 2022; Bamber et al. 2024) (~ 1 to ~ 6.5; Fig. 6c,d; brown squares and triangles), suggesting a lower number of vesicles with less tortuous connections than the Plinian case. This difference in *m* may result from differences in crystallinity, as clasts from basaltic Plinian activity have a high microlite content (Sable et al. 2006; Costantini et al. 2010; Bamber et al. 2020, 2022). More tortuous outgassing pathways can be produced by crystallisation, as bubbles deform to accommodate the rigid crystalline network (Valdivia et al. 2022), reducing magma permeability as the greater path length and curvature increases flow resistance from viscous and inertial effects (Rust and Cashman 2004). Our lapilli samples show values of *m* that are at the lower end of the range for Plinian activity (Fig. 6c, d; dark brown squares and triangles), which correspond to more crystal-poor samples.

Instead, the average *f*_*tb*_ calculated for our samples from the Tajogaite eruption (≥ 0.28) is higher than the range in *f*_*tb*_ (Fig. 6a, b) calculated for clasts of basaltic fountaining and Plinian activity (≤ 0.27) (Bamber et al. 2024). This suggests the presence of wider pore throats in our samples that might have facilitated outgassing (Polacci et al. 2008; Burgisser et al. 2017; Baker et al. 2019). This assumption is also confirmed by analysis of the throat size distribution (Fig. S4 in Online Resource 3, and Online Resource 5). For our lapilli samples, we obtained an average minimum flow path aperture of approximately 40 μm, which is higher than that obtained by Degruyter et al. (2010) for tube and frothy pumices (23 and 26 μm, respectively) belonging to the explosive Plinian eruption of the Kos Plateau Tuff. Indeed, a wider flow aperture may facilitate outgassing and, in turn, favour less explosive volcanic activity.

Decompression rates estimated for the Tajogaite eruption (calculated from the average magma ascent rates and assuming a magmastatic pressure) ranges between 10^−3^–10^−2^ MPa s^−1^ for fountaining activities, ~ 10^−3^ MPa s^−1^ for Strombolian eruptions, and ~ 10^−4^ MPa s^−1^ for ash-jet eruptions. These values are several orders of magnitude lower than those for highly explosive basaltic Plinian eruptions (~ 0.1–1 MPa s^−1^, Arzilli et al. 2019; Bamber et al. 2022), whilst they are in agreement with the weak fountaining behaviour and with the low-end member for explosive basaltic eruptions as reported by La Spina et al. (2021). Overall, our results suggest that although the tephritic/basanitic compositions of the Tajogaite eruption may result in high bubble nucleation rates, due to the small decompression rates involved during magma ascent, there may be sufficient time to develop more efficient outgassing channels with wider and less tortuous connections, facilitating outgassing. A combination of low ascent velocities (< 1 m s^−1^) and permeability development allows gas bubbles to decouple from the melt, move and coalesce, increasing the likelihood of a Strombolian or effusive eruption (Parfitt and Wilson 1995; Polacci et al. 2008). On the contrary, if low permeability and gas–melt coupling are maintained during ascent, magma fragmentation may occur once the magma is ejected from the vent or at shallow depth within the conduit, such as in the case of lava fountain (e.g. Gonnermann and Manga 2013; Thivet et al. 2020) and ash-rich jet eruptions (e.g. Edwards et al. 2020; Namiki et al. 2021), respectively. Strombolian activity may be also generated without the formation of permeable pathways, as indicated by Colombier et al. (2021). Indeed, low values of connected porosity at depth can favour extensive bubble coalescence (particularly at low magma ascent velocity), which may result in the formation of large gas slug. Although our results indicate low porosity from sample related to Strombolian activity (0.3–0.4), connectivity is greater than 0.95, which suggests that the formation of some permeable pathways were still occurring during this type of eruption.

### Do the dynamics of magma ascent influence the pore network properties observed in natural samples?

By investigating the 2021 Tajogaite eruption, an eruption with a variation in explosivity over its duration, we have the possibility to investigate the evolution of the magma pore network parameters through time. The transition from a tephritic to a basanitic composition over the course of the eruption (Fig. 3) may have influenced the properties of the magma, and consequently, also the *m*, *f*_*tb*_, and VND. This variation may be reflected in the different eruptive styles that manifested over the course of the eruption. During the second part of the eruption, the MER and the magma ascent rate increases from Strombolian to ash-jet to fountain activity. In the case of Strombolian activity, gas is able to separate more efficiently from the magma (lower VND, lower *m*). However, as the intensity increases (from ash-jet to fountain activity), the gas–melt coupling also increases (higher VND, higher *m*). Due to the low viscosity of basaltic magma, permeable channels are able to deform over time. For a slower magma ascent rate, there may be sufficient time for these channels to realign along the vertical direction (i.e. with lower *m*), favouring outgassing and further reducing the ascent rate, creating a positive feedback. Instead, a faster magma ascent rate may not provide enough time for permeable channels to realign along the vertical direction, favouring gas-magma coupling. Further studies are required to better constrain the influence of magma ascent dynamics on the pore network structure for low-viscosity magmas and the timescale over which these processes may occur. However, since magma is able to deform during ascent, it is reasonable to assume that also the internal pore network can deform. Therefore, permeability should be seen as a dynamic property of magma. As the permeability of solidified samples does not necessarily represent the permeability of magma in the conduit (La Spina et al. 2017), due, for example to post-fragmentation modification, we acknowledge that the pore network parameters and magma permeability estimated using volcanic samples should be interpreted as a snapshot of magma at the time of quenching.

## Conclusions

By using X-ray computed microtomography, we quantified the 3D pore network of basanitic/tephritic lapilli clasts from the 2021 Tajogaite eruption (Canary Islands, Spain). Our findings reveal that variations in texture and pore network properties can be linked with the changes in eruptive style observed throughout the eruption, from fountain activity to Strombolian and ash-rich jets. Notably, differences in vesicle shape, porosity, connectivity and, to a lesser extent, the throat-pore-size ratio, show a clear difference across our sample suite, reflecting changes in outgassing conditions. For Strombolian activity, the lower vesicle number density and tortuosity factor suggests that there are fewer vesicles and that the channels which connect them are less tortuous than those for fountain and ash-rich jet activity, favouring a lower degree of gas–melt coupling and thus, more efficient outgassing. Instead, for lava fountain and ash-rich jet activity, the higher vesicle number density and tortuosity factor suggest a higher number of vesicles connected by more tortuous channels, promoting some degree of gas–melt coupling and thus, less efficient outgassing. Moreover, for ash-rich jets, the lower throat-pore size ratio suggests the presence of narrower channels that favour a greater degree of gas–melt coupling with respect to fountain activity, leading to magma fragmentation. Comparing our data with existing literature, we find that the VNDs, tortuosity and tortuosity factor obtained for our samples are comparable with those estimated for crystal-poor basaltic Plinian samples and fountain activity. Instead, the throat-pore size ratio calculated for our samples (≥ 0.28) shows higher values compared to samples of highly explosive Plinian or high intensity fountain activity (≤ 0.27). This suggests that the tephritic/basanitic compositions of the Tajogaite eruption may result in high bubble nucleation rates, but there is more time during magma ascent, allowing the development of more efficient outgassing channels with wider and less tortuous connections, thus facilitating outgassing.

The 2021 Tajogaite eruption represents an important case study to examine the interplay between ascent dynamics and pore network parameters (VND, tortuosity factor and pore-throat size ratio) for low-viscosity magmas.

## Supplementary Information

Below is the link to the electronic supplementary material.

Supplementary file1 Online Resource 1 Supplementary text for Methods section (PDF 291 KB)

Supplementary file2 Online Resource 2 Supplementary tables (XLSX 22 KB)

Supplementary file3 Online Resource 3 Supplementary figures (PDF 1295 KB)

Supplementary file4 Online Resource 4 Vesicle Size Distribution analyses (XLSX 1383 KB)

Supplementary file5 Online Resource 5 Throat Size Distribution analyses (XLSX 680 KB)

## Data Availability

The authors declare that the experimental and analytical data supporting the findings of this study are available within the article and its Supplementary Information.
