# Supplementary material for: Linking eruptive style with pore network geometry in tephritic/basanitic tephra from the 2021 Tajogaite eruption (Canary Islands, Spain)
Source: Bull Volcanol. 2025 May 30;87(6):49. doi: 10.1007/s00445-025-01833-0 (PMC12125037; doi:10.1007/s00445-025-01833-0)
Supplement: Supplementary file 1 — Supplementary file1 Online Resource 1 Supplementary text for Methods section (PDF 291 KB) [file 445_2025_1833_MOESM1_ESM.pdf]

Online Resource 1 for

Linking eruptive style with pore network geometry in tephritic/basanitic tephra from the 2021 Tajogaite eruption (Canary Islands, Spain)

Barbara Bonechi[1*], Emily C. Bamber[2], Margherita Polacci[1], Fabio Arzilli[3], Giuseppe La Spina[4], Elisa Biagioli[1], Jorge E. Romero[5], Jean-Louis Hazemann[6], Richard Brooker[7], Robert Atwood[8], Mike Burton[1]

1: Department of Earth and Environmental Sciences, The University of Manchester, Manchester, United Kingdom of Great Britain and Northern Ireland

2: Institute of Science, Technology and Sustainability for Ceramics (ISSMC), National Research Council (CNR), Faenza, Italy

3: School of Science and Technology, Geology Division, Camerino, Italy

4: Istituto Nazionale di Geofisica e Vulcanologia, Osservatorio Etneo, Catania, Italy

5: Instituto de Ciencias de la Ingeniería, Universidad de O'Higgins, Rancagua, Chile

6: Université Grenoble Alpes, CNRS, Grenoble INP, Institut Néel, Grenoble, France

7: School of Earth Sciences, University of Bristol, Bristol, United Kingdom

8: Diamond Light Source, Harwell Science and Innovation Campus, Harwell, Oxfordshire, United Kingdom

Corresponding author: barbara.bonechi@manchester.ac.uk

Supplementary Methods

Synchrotron-based X-ray computed microtomography: Image processing and analysis

Pre-segmentation and segmentation image processing

Following the stacking of images to produce complete sample sub-volumes in ImageJ, the image processing and analysis was completed using the commercial software Avizo (v. 2019.1; Thermo Fisher Scientific, USA). Firstly, for all samples, filters were required to segment the vesicles from the surrounding matrix glass to preserve, as much as the pixel size used permitted (>15 µm in thickness), the thin glass films separating them, and to reduce the possibility of overestimating the connected porosity and connectivity (Shea et al. 2010). Edge-preserving filters were applied, to preserve the boundaries separating features of interest within the sample. First, the 3D *median filter* with a local neighbourhood of 26 voxels and 3 iterations was applied; followed by the 3D *bilateral filter,* using a kernel size of 9 (*x*, *y*, *z*) and a similarity of 20 in bilateral mode. Finally, the 3D *non-local means filter* was applied to the output of the bilateral filter, using a spatial standard deviation between 5 and 10, and an intensity standard deviation of 0.2. The search window was 10 voxels and the local neighbourhood considered 3 voxels.

Quantitative image analysis

Quantitative image analysis was performed using algorithms available in Avizo, following the approach of Bamber et al. (2024). The *volume fraction* algorithm was used to calculate the volume of the pore space using the segmented binary images as the input. The *labelling* algorithm was then used on the binary images to identify connected pores within the sample. The *axis connectivity* algorithm was then applied to the output of the *labelling* algorithm, producing an image which contained all connected paths within the sample volume. Both the *labelling* and *axis connectivity* algorithms considered a local neighbourhood of 26, where the local neighbourhood defines how pixel connections are evaluated in a 3D space. With this 26-connected neighbourhood, the algorithm treats all voxels that touch the current voxel in any direction—whether by face, edge, or corner—as part of the same connected group. This approach ensures that connectivity is assessed in the most inclusive way possible within a 3D space. The *volume fraction* algorithm was then used on the output of the *axis connectivity* algorithm to provide the volume of the connected pore space. Smoothing of the traced spatial graph was performed (10 iterations) with a smoothing coefficient of 0.5 and a value of 0.25 regarding the influence of the initial coordinate on its new position.

To calculate vesicle number densities (VND) and size distributions (VSD), we used the *separate objects* algorithm in Avizo to separate connected vesicles. We tested multiple marker

extent values, as the degree of separation significantly impacts the calculated VND values and VSDs. A marker extent of 1 (the lowest available in Avizo) proved to be the most effective in separating the smallest vesicles while preserving the volume of larger ones. Increasing the marker extent (starting from 2 onwards) instead, led to the preservation of vesicle clusters (Fig. A1), as the software considered a larger number of touching voxels when defining connected objects. By comparing the results of the algorithm with the original images, we determined that a marker extent of 1 provided the best separation of vesicle clusters.

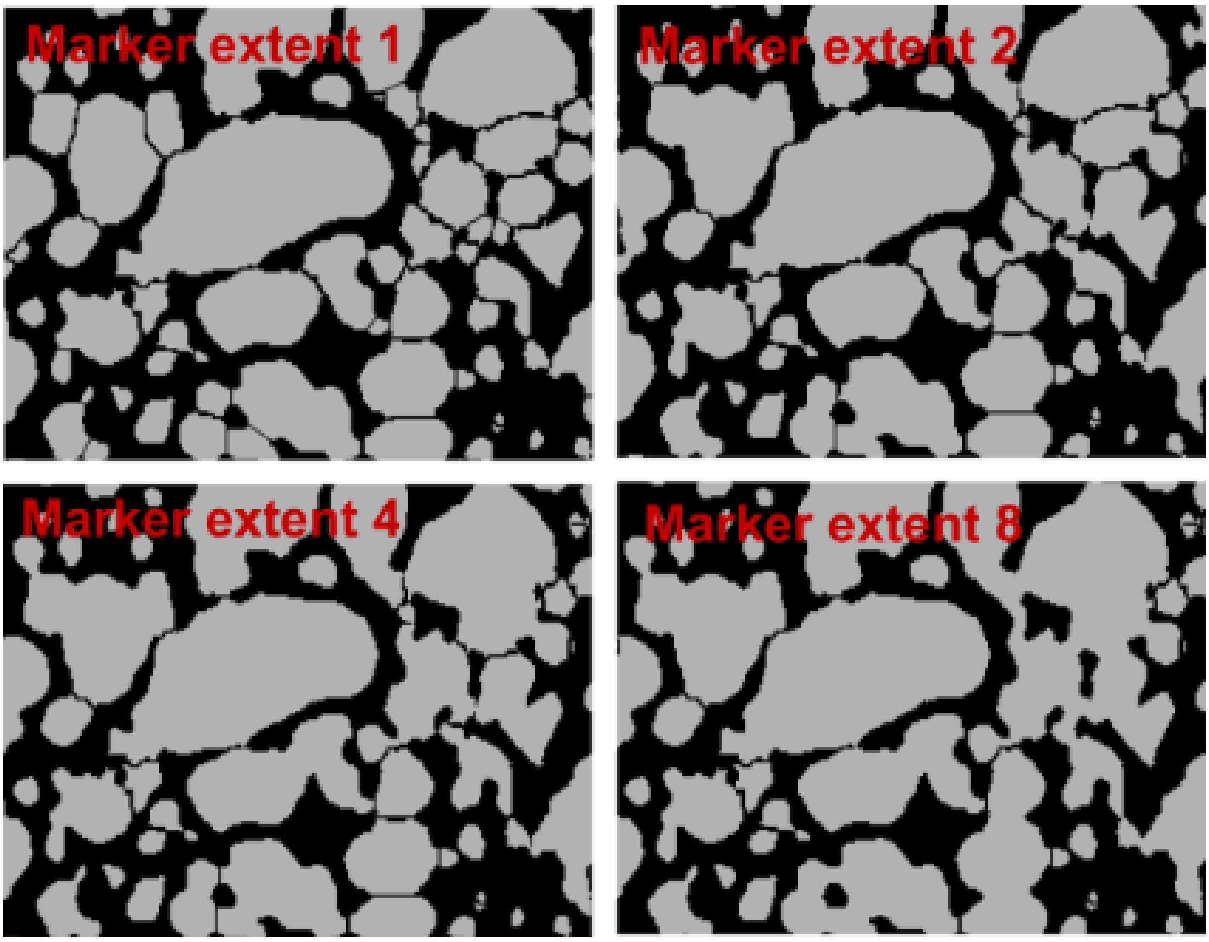

Figure A1. Slices showing the results of different marker extent values applied in the *separate objects* algorithm in Avizo. From a marker extent of 2 onwards, vesicle clusters become apparent.

To assess the impact of the separation algorithm on calculated volume number densities (VNDs) and volume size distributions (VSDs), we conducted a comparative analysis where vesicles were manually separated using Avizo. The results obtained from manually separating the vesicles were compared with those obtained using the *separate objects* algorithm in Avizo. The differences observed were minimal, with variations only in the second decimal place (see

Table A1). Since these variations were negligible and did not impact our overall results, we opted to use the Avizo *separate objects* algorithm to improve processing efficiency.

Table A1. Comparison of vesicle separation results obtained manually and using the Avizo *separate objects* algorithm

Sample	VND - manual separation	VND – Avizo *separate objects* algorithm	VND_m - manual separation	VND_m – Avizo *separate objects* algorithm
**22 Sept_c**	1.087×10^{12}	1.087×10^{12}	2.400×10^{12}	2.414×10^{12}
**25 Sept_c**	8.864×10^{11}	8.864×10^{11}	2.036×10^{12}	2.041×10^{12}
**26 Sept_c**	6.569×10^{11}	6.569×10^{11}	1.039×10^{12}	1.043×10^{12}
**15 Nov_c**	9.701×10^{11}	9.774×10^{11}	2.246×10^{12}	2.268×10^{12}
**16 Oct_b**	1.037×10^{12}	1.037×10^{12}	1.690×10^{12}	1.694×10^{12}

Finally, regarding the tortuosity calculation, a minimum segment length was considered due to the issue of multiple nodes being seeded within the same bubble by the skeletonization procedure (Lindquist et al. 1996). This issue can create an unrealistically high number of nodes whilst producing very short segments, leading to spurious values of the tortuosity. Tortuosity was, thus, calculated only for segments which exceeded the minimum length (100 μm), determined from careful examination of textures in the 3D sample volumes.

References

Bamber EC, La Spina G, Arzilli F, et al (2024) Outgassing behaviour during highly explosive basaltic eruptions. Commun Earth Environ 5:3. https://doi.org/10.1038/s43247-023-01182-w

Shea T, Houghton BF, Gurioli L, et al (2010) Textural studies of vesicles in volcanic rocks: An integrated methodology. J Volcanol Geotherm Res 190:271–289. https://doi.org/10.1016/j.jvolgeores.2009.12.003

Lindquist WB, Lee SM, Coker DA, Jones KW, Spanne P (1996) Medial axis analysis of void structure in three-dimensional tomographic images of porous media. J. Geophys. Res. Solid Earth 101:8297-8310. https://doi.org/10.1029/95JB03039
