# Supplementary material for: Linking eruptive style with pore network geometry in tephritic/basanitic tephra from the 2021 Tajogaite eruption (Canary Islands, Spain)
Source: Bull Volcanol. 2025 May 30;87(6):49. doi: 10.1007/s00445-025-01833-0 (PMC12125037; doi:10.1007/s00445-025-01833-0)

Supplementary figures for

Linking eruptive style with pore network geometry in tephritic/basanitic tephra from the 2021 Tajogaite eruption (Canary Islands, Spain)

Barbara Bonechi[1*], Emily C. Bamber[2], Margherita Polacci[1], Fabio Arzilli[3], Giuseppe La Spina[4], Elisa Biagioli[1], Jorge E. Romero[5], Jean-Louis Hazemann[6], Richard Brooker[7], Robert Atwood[8], Mike Burton[1]

1: Department of Earth and Environmental Sciences, The University of Manchester, Manchester, United Kingdom of Great Britain and Northern Ireland
2: Institute of Science, Technology and Sustainability for Ceramics (ISSMC), National Research Council (CNR), Faenza, Italy
3: School of Science and Technology, Geology Division, Camerino, Italy
4: Istituto Nazionale di Geofisica e Vulcanologia, Osservatorio Etneo, Catania, Italy
5: Instituto de Ciencias de la Ingeniería, Universidad de O'Higgins, Rancagua, Chile
6: Université Grenoble Alpes, CNRS, Grenoble INP, Institut Néel, Grenoble, France
7: School of Earth Sciences, University of Bristol, Bristol, United Kingdom
8: Diamond Light Source, Harwell Science and Innovation Campus, Harwell, Oxfordshire, United Kingdom

Corresponding author: barbara.bonechi@manchester.ac.uk

Fig. S1 2D images of September samples from reconstructed slices (left) and BSE images (right)

Fig. S2 2D images of October-December samples from reconstructed slices (left) and BSE images (right)

Fig. S3 Diagrams showing tortuosity vs (a) porosity and (b) $\log_{10}VND_m$

Fig. S4 Pore-throat size distributions of tephra samples of September (20 Sept, 22 Sept, 25 Sept, 26 Sept), October (16 Oct), November (15 Nov) and December (13 Dec)

Fig. S1 2D images of September samples from reconstructed slices (left) and BSE images (right)

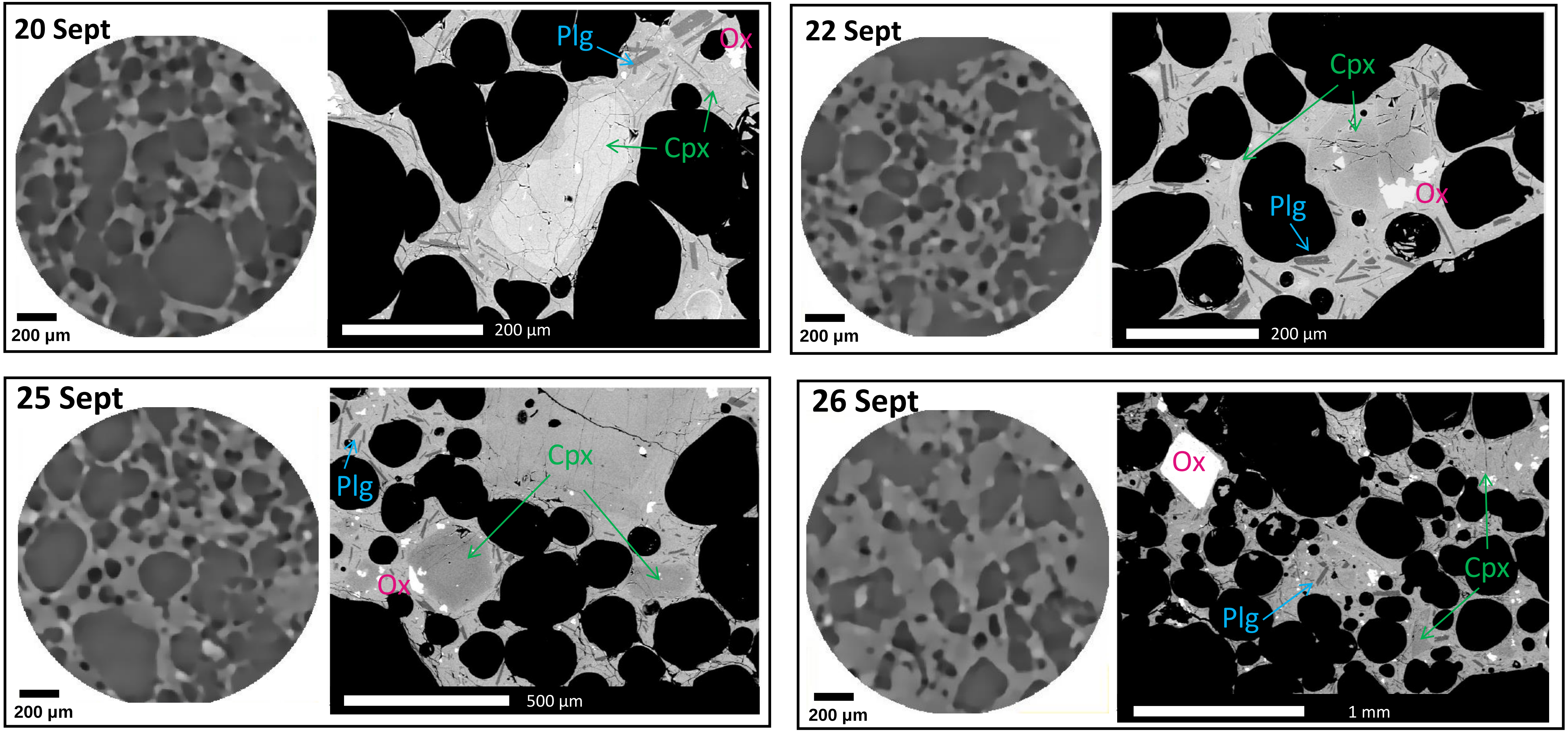

Fig. S2 2D images of October-December samples from reconstructed slices (left) and BSE images (right)

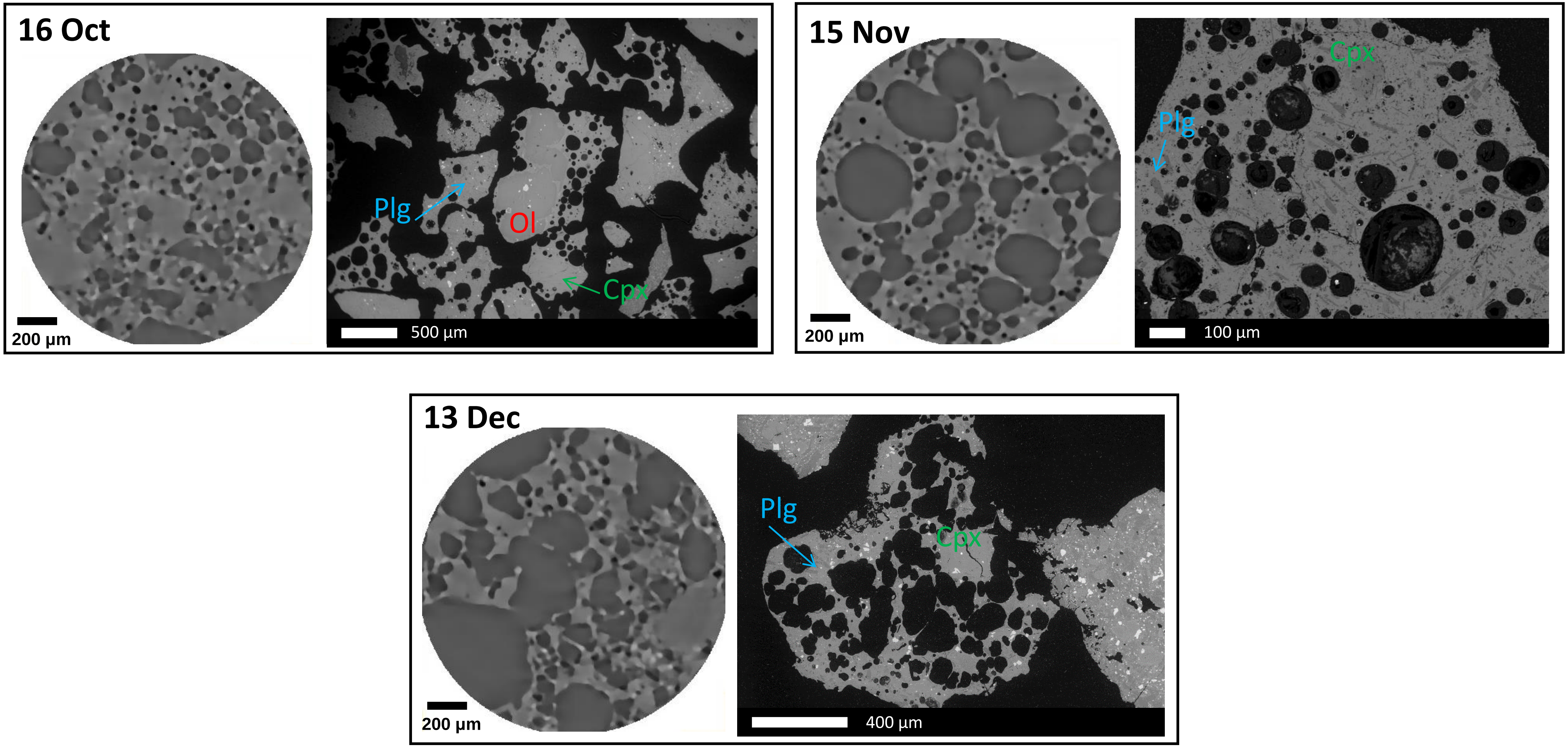

Fig. S3 Diagrams showing tortuosity vs (a) porosity and (b) $log_{10}VND_m$. Literature data as follow: B19 = Baker et al. (2019); B24 = Bamber et al. (2024). Plinian data are from Degruyter et al. (2010) and Bamber et al. (2024)

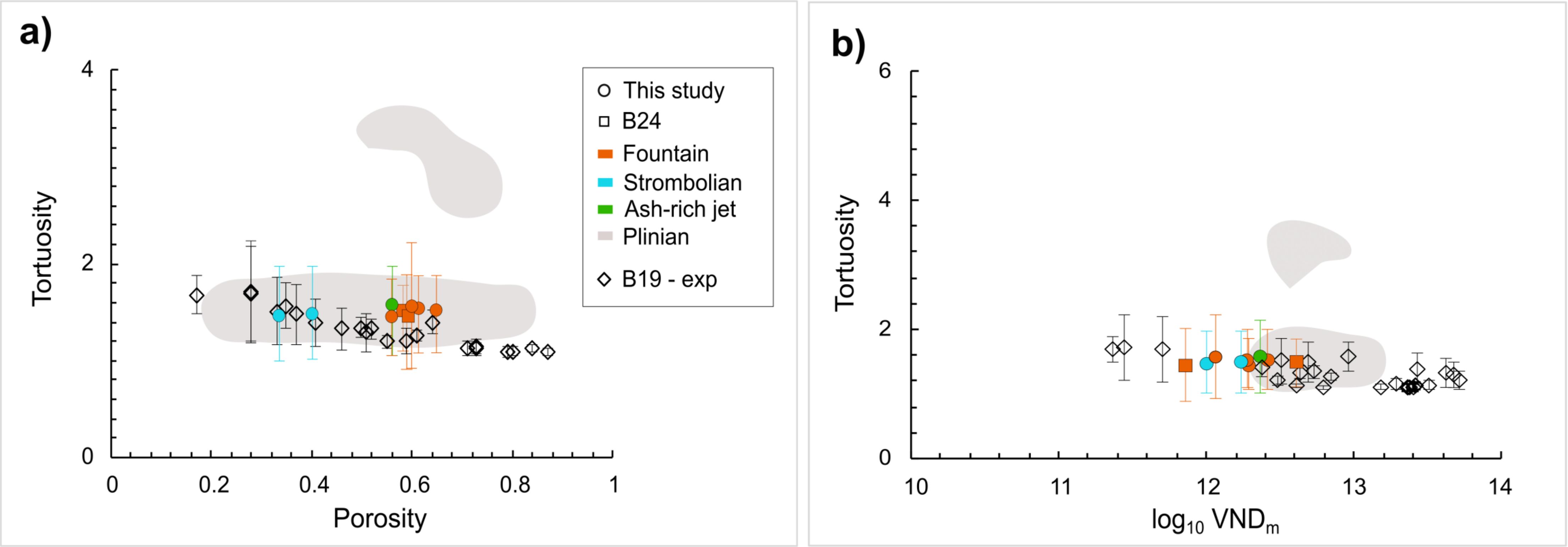

Fig. S4 Pore-throat size distributions of tephra samples of September (20 Sept, 22 Sept, 25 Sept, 26 Sept), October (16 Oct), November (15 Nov) and December (13 Dec)

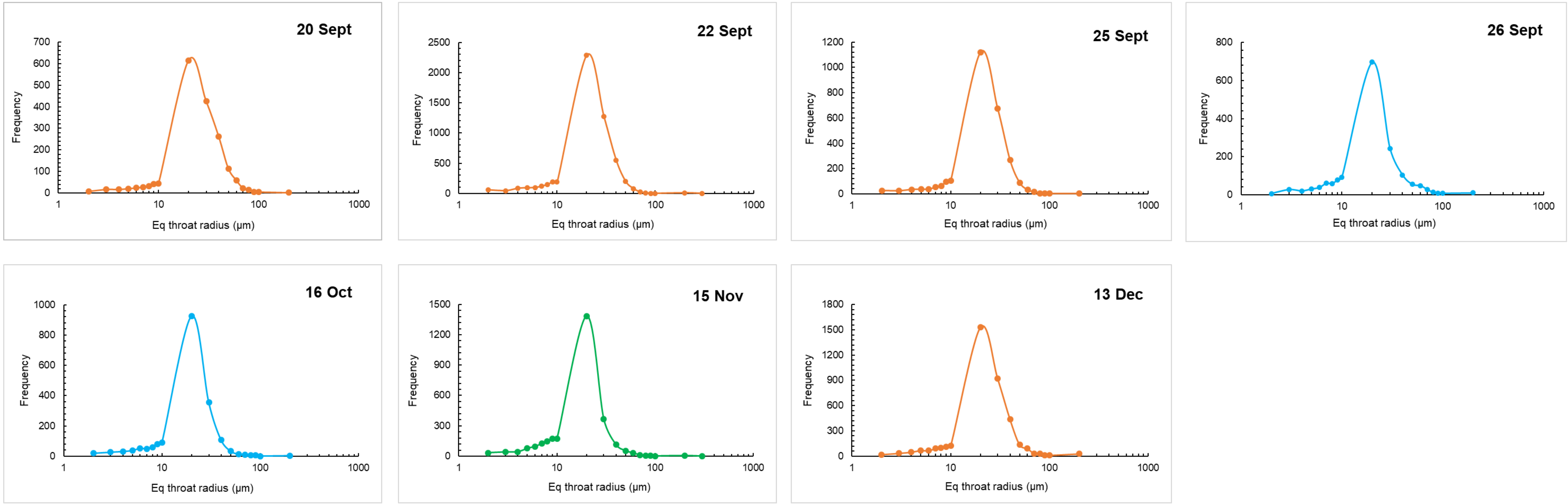

Supplement: Supplementary file 3 — Supplementary file3 Online Resource 3 Supplementary figures (PDF 1295 KB) [file 445_2025_1833_MOESM3_ESM.pdf]
